# Lysosome-mediated chemoresistance in acute myeloid leukemia

**DOI:** 10.20517/cdr.2021.122

**Published:** 2022-03-14

**Authors:** Laia Cuesta-Casanovas, Jennifer Delgado-Martínez, Josep M. Cornet-Masana, José M. Carbó, Lise Clément-Demange, Ruth M. Risueño

**Affiliations:** ^1^Josep Carreras Leukaemia Research Institute (IJC), Barcelona 08916, Spain.; ^2^Faculty of Biosciences, Autonomous University of Barcelona, Bellaterra (Cerdanyola del Vallès) 08193, Spain.; ^3^Faculty of Pharmacy, University of Barcelona, Barcelona 08028, Spain.; ^4^Leukos Biotech, Muntaner, 383, Barcelona 08036, Spain.

**Keywords:** Lysosome, chemoresistance, AML, lysosomotropic drug, lysosomal sequestration, refractory AML

## Abstract

Despite the outstanding advances in understanding the biology underlying the pathophysiology of acute myeloid leukemia (AML) and the promising preclinical data published lastly, AML treatment still relies on a classic chemotherapy regimen largely unchanged for the past five decades. Recently, new drugs have been approved for AML, but the real clinical benefit is still under evaluation. Nevertheless, primary refractory and relapse AML continue to represent the main clinical challenge, as the majority of AML patients will succumb to the disease despite achieving a complete remission during the induction phase. As such, treatments for chemoresistant AML represent an unmet need in this disease. Although great efforts have been made to decipher the biological basis for leukemogenesis, the mechanism by which AML cells become resistant to chemotherapy is largely unknown. The identification of the signaling pathways involved in resistance may lead to new combinatory therapies or new therapeutic approaches suitable for this subset of patients. Several mechanisms of chemoresistance have been identified, including drug transporters, key secondary messengers, and metabolic regulators. However, no therapeutic approach targeting chemoresistance has succeeded in clinical trials, especially due to broad secondary effects in healthy cells. Recent research has highlighted the importance of lysosomes in this phenomenon. Lysosomes’ key role in resistance to chemotherapy includes the potential to sequester drugs, central metabolic signaling role, and gene expression regulation. These results provide further evidence to support the development of new therapeutic approaches that target lysosomes in AML.

## INTRODUCTION

Relapse and refractory diseases are major clinical challenges during the management of acute myeloid leukemia (AML) patients, and they prevent an optimized response to current treatments. As relapse refers to reappearance of the disease, relapse episodes are strongly related to refractoriness, both of them leading to poor prognosis^[1]^. For the last five decades, the standard AML therapy consisted of a combination of cytarabine and an anthracycline^[2]^, and improvements in the survival rate are mainly due to optimization of the supportive care and hematopoietic cell transplantation protocols. Recently, new targeted drugs have been approved, incorporating the notion of personalised treatments for AML. Continuous assessment of newly approved drugs over time will provide valuable complete efficacy and safety data that will result in an optimal drug regime, as current clinical benefit is controversial^[3,4]^. Thus, acquisition of new biological insight in AML pathophysiology represents an unmet need necessary to expand the targetable therapeutic mechanism repertoire to overcome chemoresistance and, then, significantly improve clinical outcomes for AML patients.

Primary and secondary chemoresistance have been widely explored using conventional approaches, searching for gene mutations, chromosomal aberrations, and dysregulated signaling pathways^[5-8]^. Changes in the multidrug resistance gene family affect the intracellular concentration of drugs by either reducing the active transport into the tumor cells or increasing the efflux out to the extracellular space. Other mechanisms of action that affect the response to chemotherapy include: modifications in the chemotherapy molecular targets, preventing the pharmacologic action, increased ability to repair tumor DNA damage, defective response to proapoptotic stimuli, and changes in the tumor microenvironment^[8]^. Although several inhibitors targeting drug-resistance mechanisms have been reported, their clinical development is still under evaluation.

Oxidative phosphorylation function, metabolic plasticity, and mitochondrial adaptation contribute to chemoresistance in AML, especially towards cytarabine, as resistant AML cells rely more on mitochondrial oxidative phosphorylation and less on glycolysis^[9,10]^. Increased mitochondrial mass, mitochondrial membrane potential, reactive oxygen species production, and a characteristic gene signature associated with oxidative phosphorylation are hallmarks of chemoresistance AML cells^[9,11]^. Indeed, inhibition of oxidative phosphorylation induces chemosensitivity^[9,11-13]^; and mitochondrial oxidative phosphorylation and respiratory capacity correlate with a better response to cytarabine treatment in AML cells^[11]^. This metabolic reprogramming might have an important therapeutic implication and metabolic vulnerabilities might be exploited pharmacologically.

Closely related to mitochondria, lysosomes have attracted special interest in oncology due to their growing importance in transformation processes. The traditional view of lysosomes has been challenged by the recognition that lysosomes are not only “degradative organelles”, but also metabolic sensors and regulators, becoming legitimated as intracellular signaling hubs^[14]^. Additionally, recent findings highlight the physical and functional interaction of mitochondria and lysosomes, suggesting that this crosstalk plays a major role in metabolic regulation, based on the transfer of Ca^2+^ between organelles^[15,16]^, affecting the cellular response to treatment. In this review, the role of lysosomes in chemoresistance in AML is discussed and an overview of the potential therapeutic approaches for overcoming refractoriness in leukemia is provided.

## LYSOSOMES

Lysosomes were first described in the 1950s as organelles responsible for the degradation of biological macromolecules from extra- and intra-cellular origins^[17]^. Their central role in cellular recycling and homeostasis was revealed later when autophagy was discovered^[18,19]^. Recent discoveries confirm lysosomes as crucial modulators of cell homeostasis, regulating both cellular metabolism and clearance (reviewed in Ref.^[20]^).

Structurally, lysosomes are acidic organelles surrounded by a phospholipid bilayer. The acidic lumen is maintained by vacuolar-type H+ ATPase (V-ATPase) on the lysosomal membrane^[21]^. Other key proteins on lysosomal membrane are: lysosome-associated membrane protein, soluble N-ethylmaleimide-sensitive factor activating protein receptors, toll-like receptors, and mammalian target of rapamycin (mTOR)^[22]^. Luminal hydrolytic enzymes of lysosomes include proteases, sulfatases, nucleases, lipases, phosphatases, and nucleases, which degrade macromolecules.

Lysosomes belong to the endolysosomal system, a dynamic network of organelles consisting of early, late, and recycling endosomes and lysosomes. Primary lysosomes originate from the Golgi apparatus. Early endosomes formed from the plasma membrane might also progress to late endosomes and lysosomes, as a result of a maturation process. Alternatively, a contact site between lysosomes and late endosomes can be formed, followed by cargo transfer and dissociation (kiss-and-run model), or late endosomes can fuse with lysosomes, creating a hybrid organelle that subsequently evolves in lysosomes (fusion and fission process)^[23]^.

During malignant transformation, cancer cells adapt their physiological processes to sustain their intrinsic anabolic and catabolic needs. Both lysosomal mass and subcellular localization are widely changed to enable the acquisition of cancer cells’ idiosyncratic feature of uncontrolled growth. Recycling of exogenous material provides energy and key molecular components, while autophagy enhances catabolism and, consequently, energy and metabolite precursors are supplied. Nutrient sensing is tightly regulated by lysosomes, based on the activation and translocation of the mTORC1 complex to the lysosome membrane, enhancing lipid catabolism under starving condition in transformed cells (reviewed in Ref.^[24]^).

### Lysosomes in AML

During leukemogenesis, AML cells increase their lysosomal mass, although their number is not significantly affected^[25]^. As AML relies on fatty acids for energy supply, lysosome-dependent fatty acid oxidation rate is higher, inducing an augmented lysosomal mass to support this process^[26]^. The gene network that regulates the lysosomal biogenesis is also upregulated, similarly to the expression of key lysosomal enzymes^[27-37]^. Indeed, the lysosomal matrix enzyme activity is enhanced in AML, as compared to healthy myeloid cells, probably due to an increase in the quantity of enzymes and the influx rate^[38]^. As a consequence of these lysosomal changes, AML cells contain fragile lysosomes due to destabilization of the lysosomal limiting membrane and lower pH.

V-ATPases play both direct and indirect roles in the control of cellular signaling. Growth, survival, and differentiation signaling pathways frequently rely on these ATP-dependent proton pumps. Control of vesicular pH by V-ATPase is essential for proper signaling by many plasma membrane receptors that traffic through the recycling networks, including Notch and Wnt^[39]^. Canonical Wnt is required for the development and maintenance of AML^[40-42]^. Inhibition of V-ATPase prevents the activating phosphorylation of the Wnt receptor upon ligand binding and dysregulates ligand-mediated internalization of the receptor^[43,44]^. Although the importance of Notch as a therapeutic target in AML is still controversial, this signaling pathway regulates proliferation and cell survival^[45-47]^. Activation of the Notch receptor induces the intracellular domain to be cleaved and translocated to the nucleus, a process requiring V-ATPase function^[48-50]^. Similarly, PI3K/Akt/mTOR signaling pathway is crucial to many physiological processes, such as proliferation, gene expression regulation, differentiation, cell death, metabolism, cell survival, and migration, and it is frequently hyperactivated in AML^[51-55]^. While the precise mechanism involved in the V-ATPase-mediated modulation of mTOR remains widely unknown, inhibition of V-ATPase represses mTOR activation^[56]^, and mTOR inhibition leads to AML cell death^[57,58]^.

### Lysosomal sequestration of drugs

Lysosomal sequestration or lysosomal trapping is an important mechanism responsible for chemoresistance acquisition^[59]^. Many chemotherapeutics used in clinics (i.e., anthracyclines, taxanes, platinum-based drugs) are lipophilic, weak-base drugs and can therefore diffuse freely across lipid membranes, including the plasma membrane and lysosomal membrane. Alternatively, lysosomotropic drugs can be actively transported by inward turned multidrug efflux transporters of the ATP-binding cassette superfamily, embedded in the lysosomal membrane (originally expressed in the plasma membrane and endocytosed into lysosomes), particularly ABCB1^[60-63]^ and ABCA3^[38]^. The acidity of the lysosomal lumen facilitates the rapid protonation of weak-base drugs, impairing their ability to cross back across lipid bilayers, resulting in their marked lysosomal accumulation and compartmentalization^[64,65]^. Chemotherapeutics sequestered in lysosomes and associated to drug resistance phenomena include tyrosine kinase inhibitors^[66-68]^, topoisomerase inhibitors^[69-71]^, antimetabolites^[72]^, alkylating agents^[73,74]^, and microtubule-targeting agents^[75,76]^. Lysosomal sequestration severely affects drug subcellular distribution, significantly reducing efficiency, since lysosomes are seldom the target sites for these chemotherapeutics, and the sequestered drugs will not reach their targets^[77]^. Therefore, higher concentrations are required to achieve therapeutically relevant concentrations, increasing side effects in patients, and promoting secondary chemotherapy resistance. Treatment with these types of drugs induces expansion of the lysosomal compartment, thereby enhancing their lysosomal sequestration capacity and further increasing chemoresistance, constituting a feedback loop^[78-80]^.

The transcription factor EB (TFEB) is the master regulator of lysosomal biogenesis, by modulating the expression of genes bearing the coordinated lysosomal expression and regulation motif. In resting conditions, phosphorylated TFEB is retained inactivated in the cytoplasm by the 14-3-3 protein. Calcineurin dephosphorylates and activates TFEB, leading to its dissociation from 14-3-3 and subsequent translocation to the nucleus. mTOR phosphorylates (and inactivated) TFEB, enabling its binding to 14-3-3 in the cytoplasm. The activity of calcineurin is modulated by the release of Ca^2+^ from the lysosomes through the lysosomal Ca^2+^ transporter mucopilin (MCOLN1)^[81]^. mTOR can be inhibited by raising the pH, as lysosomotropic drugs do^[82]^. Activation of TFEB induces lysosomal biogenesis which increases lysosomal sequestration capacity and exerts a feedback loop.

Clearance of chemotherapeutics sequestered in lysosomes might also provide an additional chemoresistance mechanism. Exocytosis has been proposed as the preferred process^[83]^, as drug accumulation induces an increase of pH, leading to an activation of exocytosis^[84,85]^. Moreover, drug sequestration-induced TFEB activation partially results in the induction of lysosomal exocytosis and clearance of lysosomal content outside the cell^[86,87]^. However, once drugs have been released to the extracellular space, they can rediffuse back into the cells, making the exocytosis-mediated chemoresistance a controversial process that requires further clarification.

The anthracyclin daunorubicin (DNR), a backbone chemotherapeutic agent in first-line treatment of AML, displays physicochemical features compatible with lysosomal trapping. Early studies demonstrated that DNR intracellular distribution depends on drug treatment response. Sensitive AML cells preferentially accumulate DNR in the nucleus, where the pharmacology effect is exerted. In contrast, DNR-resistant AML cells tend to sequester DNR in lysosomes^[88]^. Indeed, expanded lysosomes are observed in response to DNR treatment, as well as a diminished nuclear drug uptake, exhibiting a 2.5/3-fold less DNR concentration in nucleus in resistant versus sensitive AML cells^[69]^. In AML, the relevance of ABCB1 in the active transportation of DNR into lysosomes is limited^[60,69]^, in contrast to other solid tumor cells^[62,63,89]^. Instead, in AML, DNR is actively influxed by lysosomal ABCA3, a transporter upregulated in chemoresistant patients and correlated with poor prognosis^[37,38]^.

## LYSOSOME-BASED THERAPEUTIC APPROACHES

**Figure 1:**
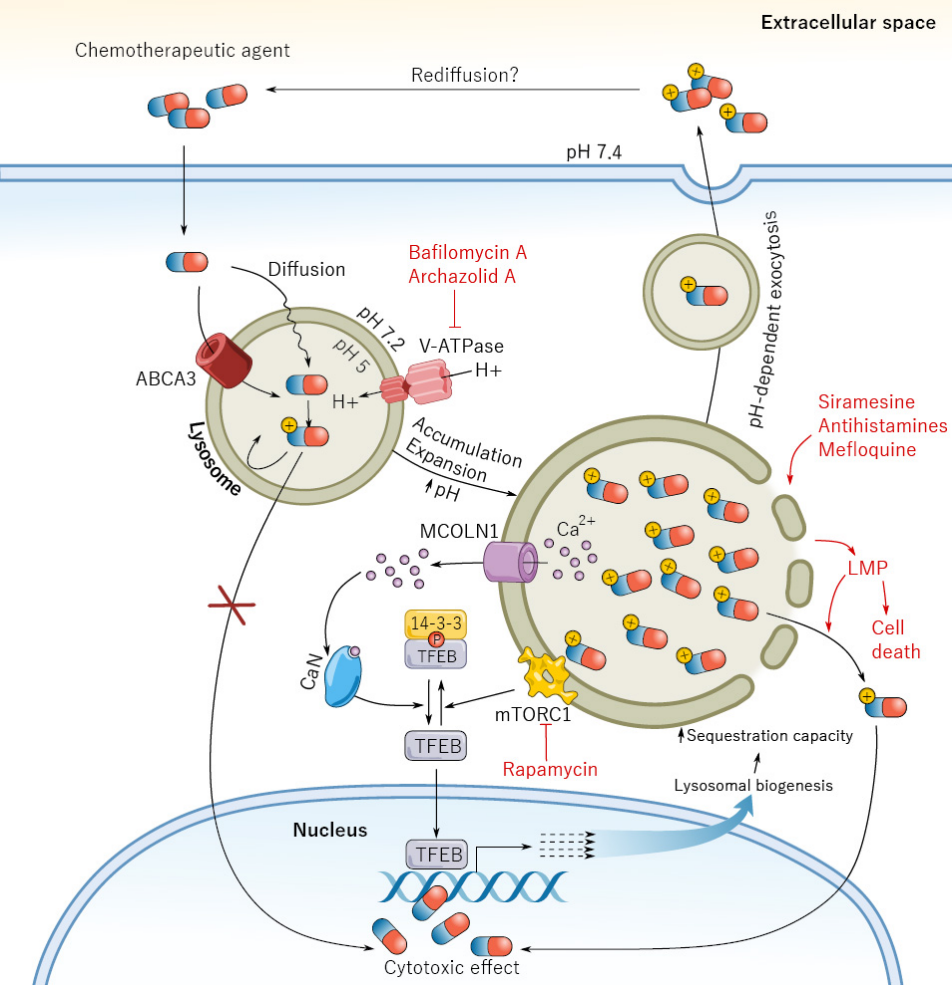
Mechanisms of lysosomal-mediated chemoresistance in acute myeloid leukemia (AML) at a glance. Most chemotherapeutic agents get readily sequestered in lysosomes upon entry in AML cells, causing a remarkable expansion of the lysosomal compartment. Lysosomal expansion is accompanied by an increase in pH, inducing exocytosis and, consequently, clearance of chemotherapy from cells. Both mechanisms prevent chemotherapeutic agents from directly interacting with their molecular targets, commonly located in the nucleus. To revert the undesirable sequestration, two main strategies have been proposed, namely, increasing lysosomal pH by inhibiting V-ATPase or pharmacologically inducing lysosomal membrane leakiness, thus releasing chemotherapeutics and additionally eliciting lysosomal-dependent cell death. Conversely, mTORC1 inhibition contributes to lysosomal biogenesis and sequestration capacity, a mechanism that has been traditionally overlooked in translation of mTORC1 inhibitors and that could partly explain their clinical failure. ABCA3: ATP binding cassette subfamily A member 3; CaN: calcineurin; LMP: lysosomal membrane permeabilization; MCOLN1: mucolipin TRP cation channel 1; mTORC1: mammalian target of rapamycin complex 1; TFEB: transcription factor EB; V-ATPase: vacuolar ATPase.

The key contribution of lysosomes to chemoresistance raises increasing interest in lysosome-targeting therapies to either sensitize tumor cells to current approved chemotherapy or as new pharmacologic approaches. The transformation process itself affects the integrity and size of lysosomes, increasing their fragility. Sphingolipid metabolism alterations are also often found in cancer cells, leading to changes in the lysosomal membrane function and structure^[90]^. Due to an increased metabolic demand, cancer cells upregulate their lysosomal function, resulting in an augmented lysosomal mass^[91]^. Accumulation of lysosomotropic drugs in cancer cells destabilizes lysosomes, causing their failure and eventually activating cell death program. However, healthy lysosomes are fully functional and display compensatory mechanisms that prevent fatal damage. Consequently, a wide therapeutic window is found due to these differences in fragility of lysosomes in cancer cells *vs.* healthy cells. Several strategies have been explored, including lysosomal destabilization. Lysosomotropic compounds can accumulate in lysosomes, causing lysosomal membrane-cell permeabilization, release of cathepsins, and consequently, activation of the cell death program^[92]^. Using different screening approaches, the anti-malaria drug mefloquine^[25]^, cationic amphiphilic antihistamines^[93]^, and σ2 receptor agonist siramesine^[94]^ were described as lysosomal disruptors in AML cells and sensitizers to approved chemotherapeutics. Mefloquine disturbs the lysosomal membrane of AML cells, allowing the release of cathepsins to the cytoplasm and inducing apoptosis^[25]^. Cationic amphiphilic antihistamines simultaneously disrupt both lysosomes and mitochondria, based on their physico-chemical properties, inducing both apoptosis and autophagy^[93]^. Both the specific cationic amphiphilic antihistamines and mefloquine spare healthy blood cells, confirming the differential effect of lysosomal disruptors in AML and the existence of a preclinically-validated therapeutic window. However, reprofiling of these drugs to AML is difficult due to their pharmacological profile, and no clinical trials have been successfully completed. Medicinal chemistry programs are expected to be necessary to achieve clinically suitable new compounds. Nevertheless, to date, targeting lysosomal integrity is the most promising therapeutic approach to overcome lysosome-mediated chemoresistance in AML [Figure 1].

Accumulation of chemotherapeutics in lysosomes heavily depends on lysosomal lumen pH. Moreover, in resistant AML cells, the pH gradient between the lysosome and cytosol is higher^[95]^. Treatment with V-ATPase inhibitor archazolid A induces cell death in leukemic cells, both T-cell acute leukemia and acute myeloid leukemia^[96]^. Similar results were obtained with bafilomycin A, another V-ATPase inhibitor, although the mechanism of action responsible for this pharmacological effect is still controversial^[97]^, as bafilomycin A is unable to resensitize cytarabine-resistant cells^[98]^. However, the preclinical data suggest that the therapeutic window was narrow, and their clinical significance might be limited.

As for further lysosome regulators, rapamycin and other mTORC1 modulators, have shown promising results in preclinical assays, specially in combination therapies^[52]^. However, mTORC1 regulates key processes implicated in cellular metabolism and growth. The complexity and broadness of the mTOR signaling networks increase the risk of toxicity due to off-tumor on-target effects, as the therapeutic window is narrow, if existent^[99]^. Besides, accumulating evidence suggest that mTOR is not the only specific molecular target for rapamycin. A quantitative chemical proteomics approach has revealed that the rapamycin targetome is extensive, including Stat3, an ubiquitous secondary messenger^[100]^. In consequence, the efficacy of mTORC1 modulators in clinical trials is limited^[101-104]^, probably due to its conserved function of mTOR complex in homeostasis mechanisms in all cell types, preventing their further clinical development [Table 1].

**Table 1:** Summary of the main lysosome-associated chemoresistance mechanisms and therapeutic approaches

CHEMORESISTANCE MECHANISMS RELATED TO LYSOSOMES			
**Mechanism**	**Cause**	**Effect**	**Solution**
Drug sequestration^[59]^	Drug protonation due to lysosomal acidic lumen^[64,65]^	Changes in subcellular distribution^[77]^	Increase drug concentration^[78-80]^
Exocytosis^[83]^	Drug accumulation due to increased pH^[84,85]^ and TFEB activation^[86,87]^	Clearance of lysosomal drug content^[86,87]^	Drugs can rediffuse back into the cells
LYSOSOME-BASED THERAPEUTIC APPROACHES			
**Type**	**Actions**		**Examples**
Lysosomal destabilizers	Lysosomal membrane permeabilization, cathepsins release, and cell death program activation^[92]^		Mefloquine (anti-malaria drug)^[25]^, cationic amphiphilic antihistamines^[93]^, and siramesine (σ2 receptor agonist)^[94]^
V-ATPase inhibitors	Activation of cell death program^[96]^		Archazolid A^[96]^ and bafilomycin A^[98,96]^
mTOR modulators	Effect on combinatory therapies^[52]^		Rapamycin^[52]^
Antibody-drug conjugates	Release of the therapeutics coupled to the antibody^[105]^		Gemtuzumab ozogamicin^[106]^

Gemtuzumab ozogamicin (Mylotarg) is an anti-CD33 monoclonal antibody conjugated to the small molecule chemotherapy drug calicheamicin, recently reapproved for AML. Upon surface CD33 recognition, this antibody-drug conjugate is internalized and translocated to lysosomes. The acidic-labile linker is hydrolyzed in the acidic environment of the lysosome, releasing the cytotoxic drug that is exported to the nucleus, where the pharmacological effect occurs^[105]^. Thus, the effectiveness of gemtuzumab ozogamicin greatly depends on lysosome functionality. This targeted therapy was expected to represent a new paradigm in AML therapy; however, the clinical benefit is limited and severe adverse effects are found in a considerable rate^[106]^. Discrepancies between expectations and clinical efficacy may be explained based on the lysosomal impairment in AML cells, partially due to the hyperactivation of PI3K/Akt signaling pathway^[51,53,54]^. A direct correlation between lysosome function and gemtuzumab ozogamicin-induced cytotoxicity was observed and forced activation of lysosomes led to a synergistic effect with gemtuzumab ozogamicin^[106]^, demonstrating that these lysosomal-dependent conjugate approaches used as monotherapy may be of limited interest in AML.

## CONCLUSION

Refractory and relapse disease are still the main clinical challenges faced in AML. Although new drugs have been approved in the last years, treatment failure and resistance mechanisms remain a major problem in patient management. To date, none of the therapeutic strategies designed to overcome chemoresistance has succeeded in clinics. The identification of lysosomes as key organelles in resistance acquisition opened a new research field and provided new avenues to explore in order to revert the resistant phenotype. The leukemic transformation process results in an augmented metabolic demand, associated with the upregulation of the lysosome function. Consequently, increase in lysosomal mass, pH, and enzymatic activity is induced. Lysosomotropism of several chemotherapeutics enable their sequestration in the lysosomes, becoming “drug-safe house” compartments and reducing their cytotoxic effect in molecular targets. As a consequence of the lysosomal changes induced during leukemogenesis, AML lysosomes are more fragile than those found in healthy cells, with a preclinically demonstrated safe therapeutic window. Developing new drugs that target leukemic lysosome integrity may sensitize AML cells to conventional chemotherapeutics or even constitute a new pharmacological lysosome-centred strategy for relapse and refractory AML patients.

## DECLARATIONS

### Copyright

© The Author(s) 2022.
